# Effect of 6-week BFRT combined with IASTAM therapy on international standard dancers with chronic ankle instability

**DOI:** 10.3389/fphys.2024.1417544

**Published:** 2024-09-26

**Authors:** Yang Liu, Ying Wang

**Affiliations:** Graduate School, School of Arts, Wuhan Sports University, Wuhan, China

**Keywords:** chronic ankle instability, international standard dance, dance sports, blood flow restriction training, compression training, ankle strength training, ankle balance training, instrument-assisted soft tissue mobilization

## Abstract

**Background:**

In sports dance events, athletes often face the risk of ankle injury and instability, which may have a negative impact on their training and athletic performance, and even hinder their rehabilitation process and increase the likelihood of re-injury.

**Objective:**

This study aims to observe the effects of exercise intervention (low-load ankle muscle strength training with blood flow restriction training (BFRT) equipment and balance training with blood flow restriction training equipment) combined with instrumentation therapy (Instrument-assisted soft tissue mobilization, IASTM) on ankle function, joint range of motion, and strength in sports dancers with chronic ankle instability (CAI). This study aims to provide an evidence-based approach to rehabilitation for athletes by comparing the effects of combination therapy approaches to traditional ankle strength and stability training.

**Methods:**

Forty-two subjects with ankle instability, restriction, or discomfort were selected as observation objects and randomly divided into three groups: the combined group (n = 14, blood flow restriction training combined with IASTM), the simple blood flow restriction training group (n = 15), and the conventional ankle strength and stability training group (n = 13). The intervention lasted for 6 weeks, once a week. The three groups were assessed with the Cumberland ankle instability assessment, Foot and Ankle Ability Measure (FAAM) ankle function assessment score, and ankle range of motion measurement before intervention, after the first intervention, and after 6 weeks of intervention. The ankle strength test was compared and analyzed only before and after intervention.

**Result:**

There was no significant difference in the participant characteristics of the three intervention groups. In terms of Cumberland Ankle Instability Tool (CAIT) scores, within-group comparisons showed that the scores after the first intervention and at the 6-week mark were significantly higher than before the intervention (P < 0.05). Between-group comparisons revealed that the combined intervention group had higher CAIT scores than the other two groups after the 6-week intervention. Regarding the FAAM functional scores, all three interventions significantly improved ankle joint function in patients with chronic ankle instability (P < 0.05), with the BFRT group showing significantly higher FAAM - Activities of Daily Living scale (FAAM-ADL) scores than the control group (P < 0.05). Both the combined and BFRT groups also had significantly higher FAAM-SPORT scores after the first intervention compared to the control (P < 0.05). In terms of ankle range of motion improvement, the combined intervention group showed a significant increase in ankle joint motion after the intervention (P < 0.05), particularly in the improvement of dorsiflexion ability (P < 0.05). As for ankle strength enhancement, all three intervention groups experienced an increase in ankle strength after the intervention (P < 0.05), with the combined intervention group showing a significant improvement in both dorsiflexion and inversion strength compared to the control group (P < 0.05).

**Conclusion:**

BFRT combined with IASTM, isolated BFRT, and conventional ankle strength and stability training significantly improve stability, functionality, and strength in CAI patients. The combined intervention demonstrates superior efficacy in improving ankle range of motion compared to isolated BFRT and conventional approaches.

## Introduction

In the dance realm, sports dance, also known as international standard dance, is divided into two major categories: Latin dance and Modern dance. In the daily training of these disciplines, mastering movements such as posture, footwork, turns, and rotations requires diligent practice, placing significant demands on the athleticism, leg strength, and stability of sports dancers (Liederbach et al., 2012; Kuliś and Gajewski, 2022). Consequently, athletes in sports dance often face various risks of sports-related injuries (Jacobs et al., 2017). Among these, ankle injuries are particularly prevalent due to the complexity of the ankle joint, which comprises multiple ligaments and muscles that provide support and stability to the joint (Golanó et al., 2010). However, these anatomical structures are often challenged during high-intensity dance movements. Actions like rapid turns, jumps, and twists subject the ankle joint to substantial pressure and torsion, making ligament injuries or strains common occurrences (Colombié and Ladoucette, 2023; Rice et al., 2023). Especially in dance routines that involve frequent rotation and twisting, the load on the ankle joint becomes more intense, thereby increasing the risk of injury. Additionally, sustained high-intensity dance training and performances may lead to muscle fatigue in the ankle joint, further elevating the risk of injury. Decreased muscle control ability under fatigue can cause dancers to lose balance or execute movements with incorrect posture, thereby heightening the likelihood of ankle injuries (Chui et al., 2022; Wu X et al., 2023). Among these injuries, Chronic ankle instability (CAI) is a prevalent and significant issue in sports dance (Sohl and Bowling, 1990; Simon et al., 2014). CAI is closely associated with recurrent twisting injuries during sports activities and long-term anomalies in ankle joint function (Chang et al., 2021; Bonnel et al., 2010). Its primary characteristics include reduced stability of the ankle joint, with patients often feeling insufficient stability in the joint while standing or moving, making them prone to recurrent ankle sprains during walking, sports, or other activities (Gribble, 2019; Bonnel et al., 2010).

Ankle injuries not only affect athletes’ competitive performance, but can also delay the rehabilitation process and negatively affect their careers. After the symptoms are relieved, the patient’s calf muscles are in a state of atrophy due to lack of exercise, especially the gastrocnemius muscle and the tibialis anterior muscle around the ankle (Drakos et al., 2023; Bonnel et al., 2010). Muscle weakness or incomplete ligament injury will lead to insufficient joint support, thus affecting the stability of the foot and ankle, which is one of the important factors of CAI (Miklovic et al., 2018; De Cesar Netto et al., 2023). In addition, nerve control of muscle movement may be affected, resulting in poor muscle coordination and increasing the risk of foot and ankle joint instability (Mohamadi et al., 2020; Han et al., 2022). Therefore, the strength and stability of the ankle joint after rehabilitation is particularly important, this training can not only improve the strength of the ankle joint, but also enhance its stability, laying a solid foundation for future sports and life.

Blood Flow Restriction Training (BFRT) is a unique training technique that aims to promote muscle growth and strength improvement by restricting blood flow through the use of a cuff or elastic on the extremities (Hughes et al., 2017). Initially, BFRT was mainly used for rehabilitated athletes, due to its effect of improving muscle strength while reducing load, which helps to avoid further injury in the rehabilitation process (Lorenz et al., 2021). However, with the deepening of research, it has gradually been widely used in the field of fitness and therapeutic exercise (Pignanelli et al., 2021; Wortman et al., 2021). A major advantage of BFRT is that it allows for efficient therapeutic exercise under relatively light loads, reducing the burden on joints and tendons, making it suitable for people who have difficulty with high-intensity training due to injury or other reasons (Lorenz et al., 2021). BFRT can not only be used for strength therapeutic exercise, but also for rehabilitation, improving endurance and improving sports performance, which makes it have application potential in different fields (Pignanelli et al., 2021). BFRT is integrally applied across multiple anatomical regions within rehabilitation protocols, aiming to expedite the recuperation of injured areas and augment muscular strength. It exerts direct influence on the extremities’ muscle groups, particularly around the elbow and knee joints, and indirectly targets areas such as the shoulders, hips, and buttocks (Centner et al., 2019; Bobes Álvarez et al., 2020; Constantinou et al., 2022; Liu and Wu, 2023). Empirical studies have highlighted BFRT’s efficacy in stimulating the calf muscle complex in athletes afflicted with CAI). Specifically, during low-intensity resistance exercises, there is a pronounced reduction in the calf muscle’s oxygen saturation, alongside a significant escalation in the subjective rating of muscle fatigue (Killinger et al., 2019). BFRT has been demonstrated to positively contribute to the enhancement of lower limb strength and functionality in CAI patients (Burkhardt et al., 2021; Burkhardt et al., 2021; Werasirirat and Yimlamai, 2022).

Instrument-Assisted Soft Tissue Mobilization (IASTM) is a physical therapy technique that utilizes specially designed tools, such as metal or plastic scraping boards, to assist with soft tissue problems (Ikeda et al., 2019). It is mainly used in rehabilitation medicine, sports medicine and plastic surgery to deal with tension, adhesion, pain and motor dysfunction of muscles, fascia and tendons (Lu et al., 2020; Mylonas et al., 2021; Liu and Wu, 2024). The edge design of the IASTM special tool loosens adhesions in the tissue, improving the elasticity and plasticity of the tissue. Modulate pathological areas through neural pathways to reduce pain and improve nerve function (Gunn et al., 2019). In addition, different kinds of therapeutic tools can improve the accuracy of treatment, promote blood circulation, accelerate the rehabilitation process, improve tissue elasticity, and expand the range of joint motion, with lower risks and complications compared with invasive surgery (Aggarwal et al., 2021; Kim et al., 2017; Seffrin et al., 2019). Studies have shown that IASTM can significantly improve lower limb joint function, reduce pain, and increase range of motion (Liu and Wu, 2024; Gunn et al., 2019). In recent years, studies have proved that IASTM combined with BFRTI can reduce pain in patellofemoral pain patients, improve soft tissue flexibility around the knee, and enhance lower limb muscle strength. Moreover, in terms of overall therapeutic effect, IASTM combined with BFRTI is significantly better than IASTM alone (Liu and Wu et al., 2023).

Specifically, while existing studies have demonstrated that traditional rehabilitation and surgical interventions possess certain therapeutic effects for CAI patients, these methods are often limited by factors such as prolonged recovery periods, poor patient compliance, or potential surgical risks (Valderrabano et al., 2006). Moreover, current treatment approaches may not adequately consider the comprehensive improvement of muscle strength, joint mobility, and neuromuscular control. Our study aims to fill this gap by exploring an integrated intervention model combining low-load ankle muscle strength training with BFRT and IASTM therapy, offering a potentially safer and more effective rehabilitation strategy for CAI patients.

The study is based on the hypothesis that the combined treatment of BFRT and IASTM can bring about better rehabilitation outcomes for CAI patients without increasing physical burden, by promoting muscle growth, enhancing muscle strength, and improving soft tissue function. We anticipate that this research will provide new insights and contribute to the development of personalized and comprehensive rehabilitation treatment plans for CAI.

## Research object and method

### Research object

This study developed compliant inclusion and exclusion criteria based on the International Ankle Association (Gribble et al., 2014). In order to further study the problem of chronic ankle instability and its treatment, the subjects were recruited from students majoring in sports dance in Wuhan Institute of Physical Education. Students with chronic ankle instability or recurrent sprain were eligible for inclusion. Before the experiment began, The patients were required to accept Single Leg Stance (Xue et al., 2023), Trendelenburg Test (McCarney et al., 2020), Dynamic Balance Test (Wang et al., 2021), and Anterior Drawer ankle forward test (Li et al., 2020) and Y-Balance test (Plisky et al., 2021). If the subject had positive results in two or more of the above tests (Gribble, 2019; Song et al., 2016), it was judged to be chronic ankle instability and met the experimental conditions.

In this study, a single-blind design was implemented to ensure the objectivity of the results. Participants were not informed about their group allocation, which was determined by a researcher independent of the data collection team. Assessors, who were trained prior to the study to maintain consistency in evaluation, remained unaware of the participants’ group assignments throughout the study. Data were anonymized during collection and managed through a coding system that concealed the group identities from researchers until the final analysis. These measures were taken to minimize bias and ensure the validity of the study findings. A total of 42 eligible subjects were recruited for this experiment, all of whom had signed informed consent forms. This study has been officially registered on the ClinicalTrials.gov platform (registration number: NCT06251414), and was approved by the ethical review Committee of Medical School of Wuhan University of Sport. The experiment will be conducted in the Exercise Rehabilitation Laboratory and National Fitness Center of Wuhan Institute of Physical Education. The experimental study started at 2022-09-30 and ended at 2022-11-01.

#### Inclusion criteria

1) Age Range: They are between 18 and 35 years old
2) Symptom Duration: Patients presenting with symptoms of chronic ankle instability for more than 3 months, ensuring that the symptoms are stable and meet the definition of a chronic condition.
3) CAIT Score: CAIT score of 24 or less, which assesses the severity of ankle instability.
4) Functional Screening: Successful completion of ankle function screening to ensure the presence of functional limitations.
5) Structural Examination: No evidence of joint structural lesions or congenital ankle deformities upon physical examination.
6) General Health: Participants in good overall health without severe respiratory, cardiovascular, neurological, or other systemic diseases.
7) Exercise Capacity: Adequate physical capacity to perform exercise loads of a certain intensity and duration.
8) Voluntary Participation: Voluntary participation in the study with informed consent provided.

#### Exclusion criteria

1) Surgical History: History of ankle surgery, as surgery may affect ankle structure and function.
2) Trauma History: Recent ankle trauma, such as sprains or fractures, which may influence the rehabilitation process and outcome assessments.
3) Skin Conditions: Presence of skin irritation, infection, or open wounds that could affect treatment or assessment.
4) Blood Disorders: Hematological diseases such as anemia or hypotension that may affect exercise tolerance and recovery.
5) Systemic Diseases: Severe systemic diseases, including heart, lung, or neurological diseases, that may affect physical capacity and health status.
6) Cognitive Ability: Ability to understand and follow study procedures, excluding patients with cognitive impairments.
7) Compliance: Anticipated inability to adhere to study requirements or complete the entire study period.
8) Other Conditions: Any other conditions that may affect the interpretation of study results, such as participation in other interventional studies.

### Experimental group

A randomized controlled experimental design was used in this study, and scientific simple randomization method was used to group subjects according to the time order of recruitment.

The experimental group adopted a comprehensive program of exercise intervention and physical therapy, which combined BFRT and IASTM as the core intervention measures. The intervention was conducted once a week for 6 weeks.

**FIGURE 1:**
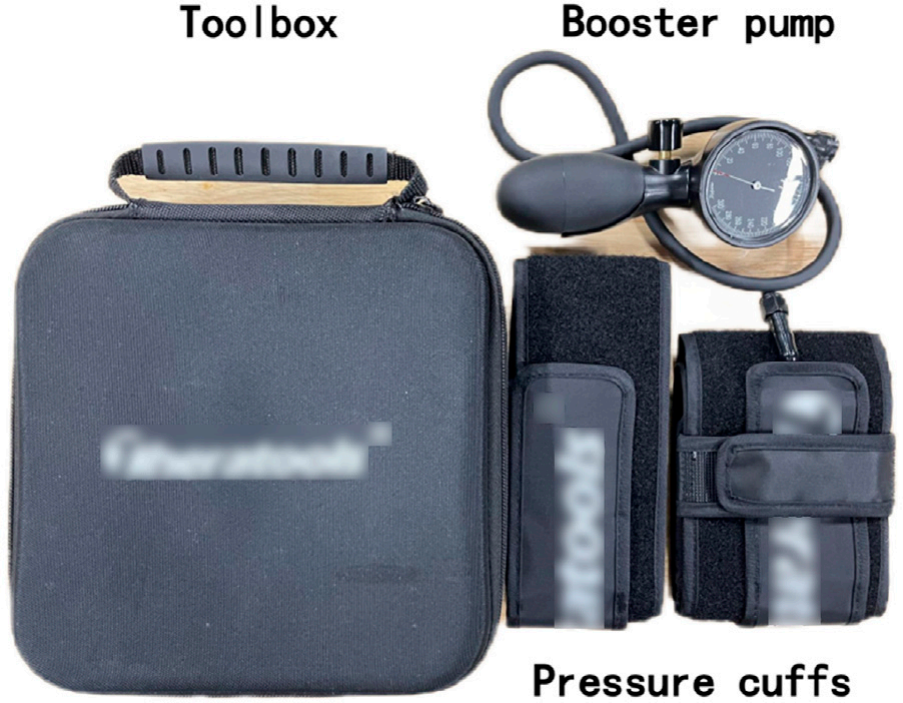
Blood flow restriction training equipment.

The BFRT device consists of a compression pump and lower limb compression cuff (Figure 1). The pressure belt is positioned at the proximal one-third of the subject’s thigh. Prior to training, the tourniquet is adjusted by pulsating compression, set at an installation pressure of 20 mmHg, and the warm-up activities are completed at this pressure value. Depending on the subject’s different lower limb dimensions and self-perception, the compression value is adjusted to 20–50 mmHg 1RM during training (Lixandrao et al., 2018), without compression during the training process. The full range of motion is supervised by a professional coach for error correction and pressure value regulation.

Based on the literature review, (Xing, et al., 2019), suggestions are made for the selection of exercise variables in blood flow restriction training. The optimal exercise variable data includes: load 20%–50% 1RM, repetition division 15–30 times/group, each exercise 50–80 repetitions (e.g., 30-15-15-15 repetitions), number of sets 3–5, rest time within sets 30–60 s (with compression), rest time between sets 5 min (without compression) (Lixandrao et al., 2018). Therefore, the exercise variables in this experiment are set according to the corresponding exercise plan on this basis. The training program followed mainly focuses on enhancing the stability of the ankle joint and the strength of the muscles around the ankle joint.

In the implementation of ankle stability training, Bosu ball (Mendez-Rebolledo et al., 2022) was used to design four core movements: one-leg support, kicking balance, plank support and squat. At the same time, in order to improve the strength of the ankle muscle group, we specially designed heel raising training, combined with elastic band resistance assistance. In response to the common problems of limited dorsiflexion and insufficient valgus strength in CAI patients, the intensity of these two exercises was increased. The above experimental groups were trained twice a week for a total of 6 weeks. The specific scheme is detailed in Table 1.

**TABLE 1:** BFRT ankle training program.

Item	Training process	Intensity (repetitions/sets)	Pressure value
Heel lift	The experimental subjects were required to wear BFRT equipment, stand on a step 10–15 cm high, maintain an upright posture, knee joints slightly bent, and toes forward. Keep your front foot on the edge of a step, your heels in the air, and your hands on a wall or chair for balance. In the centripetal phase, exhale and extend the ankle joint, contract the back of the calf muscles, and stand on your toes. The centrifugal stage inhales, flexes the ankle joint, sinks the body’s center of gravity, and elongates the muscles of the back of the calf	12-15/4-6	20–50 mmHg
Resistance to dorsiflexion of the foot	Subjects sat upright with their legs extended and wore BFRT gear. The elastic band was fixed to the back of the front foot of the subject, with both ends firmly held. In the centripetal phase, the subject exhale, dorsiflexion the ankle, pull the elastic band to exert maximum resistance, and hold for 3-5 s when the ultimate Angle is reached. During the centrifugation phase, the subject inhaled, the ankle was gradually extended, and the elastic band slowly released the tensile force and returned to the starting position	15-20/6	20-50 mm g
Resist foot valgus	Subjects sat on the BFRT gear with their legs straight. The experimenter wrapped the elastic band around the outside of the subject’s affected foot and held both ends firmly During the centripetal phase, the subject exhale and valgus the ankle, using an elastic band to generate maximum resistance, maintaining the maximum valgus Angle for 3-5 s During the centrifugal phase, the subject inhale and invert the ankle so that the elastic band slowly releases the tensile force until the ankle returns to its initial position	15-20/6	20-50 mm g
Resist foot varus	The subjects were asked to wear BFRT gear, sit, and stretch their legs naturally. Wrap the elastic band around the inside of the affected foot. In the centripetal phase, the subject exhales, synchronously pronates the ankle joint and pulls the elastic band to the maximum resistance, maintaining the ultimate Angle of pronation for 3-5 s. In the centrifuge phase, the subject inhales, turns the ankle, and slowly releases the elastic band to restore the ankle to its original position	10-12/3	20-50 mm g
Single Leg Support (Bosu)	The subject placed the Bosu ball smoothly with the sphere facing up and the plane facing down. After wearing the BFRT equipment, the subject stands on one leg on the sphere, avoiding the use of external support. While standing, bend one leg slightly and breathe deeply for balance. Hold this position for 30-60 s	30-60/3	20-50 mm g
Kick Balance (Bosu)	The Bosu ball is placed smoothly with the sphere facing up and the plane facing down. Subjects wear BFRT gear and stand on a sphere with one leg, avoiding the use of external supports. When standing, bend one leg slightly, bend the healthy side leg 90°, complete the high leg lift action, pay attention to breathing coordination. Exhale in the heart phase, raising the healthy leg to the highest point; Inhale during the centrifugal phase and slowly lower the healthy leg to the starting position	10-15/3	
Planking (Bosu)	The Bosu ball is placed smoothly with the plane facing up and the sphere facing down. Subjects wear BFRT equipment and place their legs on the Bosu ball plane. After maintaining a stable posture, complete 30-60 planks to improve balance	30-60/2	20-50 mm g
Squats (Bosu)	Subjects place the Bosu ball on the ground with the plane facing up and the sphere facing down. After wearing the BFRT equipment, place the legs on the Bosu ball plane and perform the no-load squat. Pay attention to breathing coordination when executing. Inhale while squatting, slowly bend the hips, knee flexion to below 90°, and keep the ankle joint stable. Exhale while standing, slowly extend the hips, and gradually straighten the knees to return to the initial position. Maintain ankle stability throughout	15-20/4-6	20-50 mm g

**FIGURE 2:**
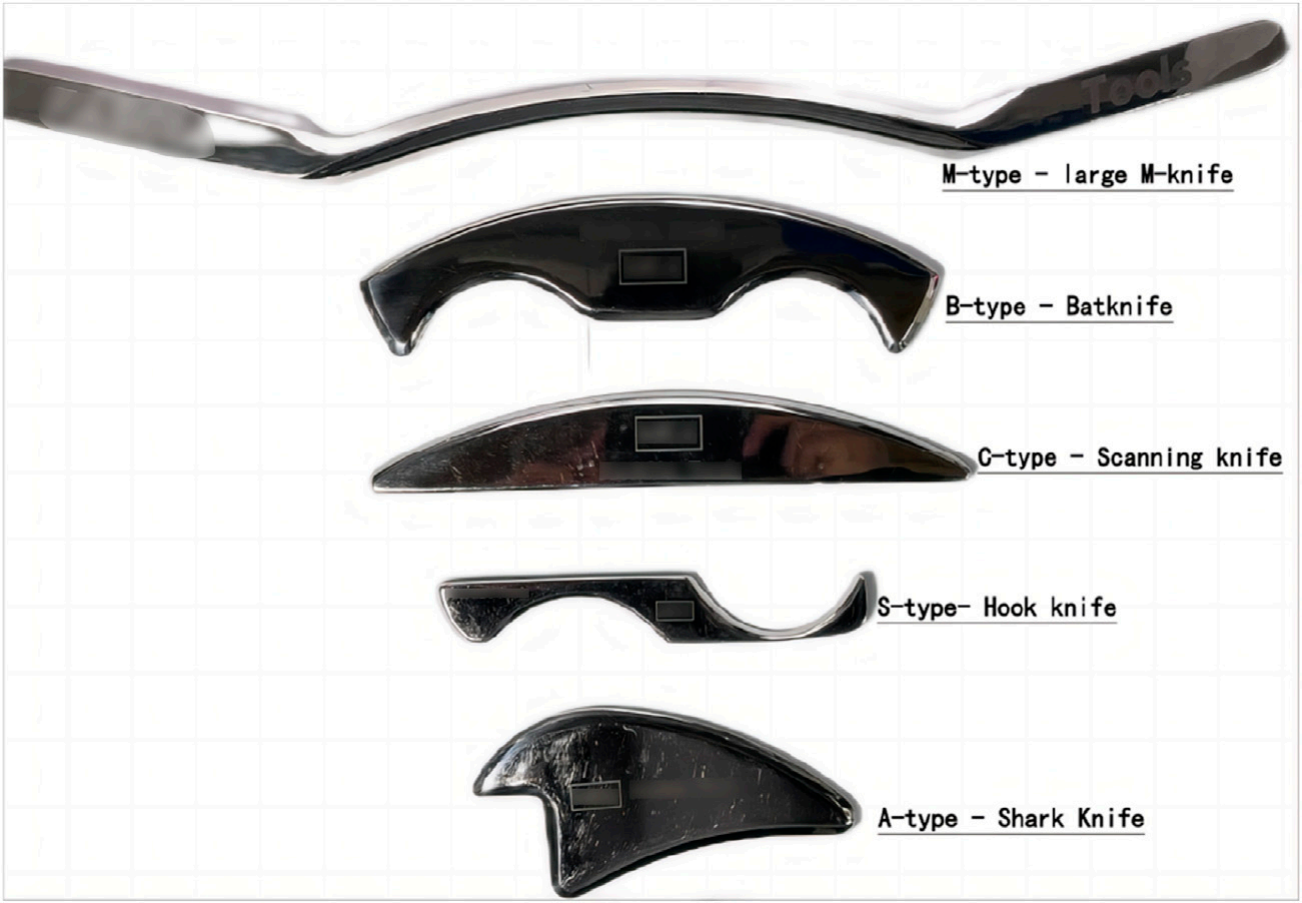
IASTM treatment tool.

The fascial knife was used as the IASTM treatment tool (Figure 2), including five types of knife: type A shark knife, type B bat knife, type C probe knife, type M large M knife and type S hook knife. These knife types have different functions and applications due to their unique shape. The IASTM intervention before BFRT is mainly to release the soft tissue around the leg and ankle joint through the physical action of a fascial knife, so as to relieve ankle pain and restore the range of motion of the ankle joint. Refer to Table 2 for detailed ankle IASTM procedures.

**TABLE 2:** Steps for IASTM operations.

Tool	Operation process	Intensity	Time (min)	Purpose
C-type—Scanning knife	Subjects should keep prone position and apply appropriate amount of fascial lubricant to the posterior or anterior side of the calf. Sliding operation was performed by applying a 45° cut Angle pressure from bottom to top and from top to bottom along the direction of the calf muscle fibers using a C-type probe	Low	1	In order to ensure that subjects can adapt to the rhythm of tool treatment, it is necessary to carefully observe and judge the calf fascia to determine the position of the densified or granular area (Weiss and Kalichman, 2021; Schilder et al., 2014; Pavan et al., 2014)
A-type—Shark Knife	A progressive and robust pressure sliding operation is implemented for the dense area or the high-resistance area where the pain trigger points (Celik and Mutlu, 2013) are located in the posterior and anterior fascia of the leg	Low-Medium	2	In order to ensure that the soft tissue in the stiff area of the lower leg recovers elasticity, while reducing or eliminating pain points, appropriate soft tissue release should be performed at rest and at maximum stretch
B-type—Batknife	During the treatment, appropriate pressure should be applied at an Angle of 45° to the treatment area on the posterior or anterior side of the leg, and sliding pressure should be applied in the direction of top to bottom and bottom to top. In the area of fascia densification or pain stimulation point, a small area of repeated pressure sliding should be performed to ensure the therapeutic effect	Medium-High	3-5	In order to achieve deep release of the calf muscle fascia, we will use concentrated and moderately increased pressure to perform the operation
M-type—large M-knife	To ensure experimental accuracy and subject safety, subjects were required to perform foot dorsiflexion and toe flexion movements continuously, reaching the limit of each movement. When the maximum Angle is reached, hold still for 3–5 s and fully stretch the muscles and fascia. At the same time, with deep breathing, enhance the muscle relaxation effect	Medium-High	5-10	In order to ensure full dynamic release of the deep muscle group of the lower leg, it is necessary to increase the intermuscular sliding and gradually restore the range of motion of the ankle joint
S-type—Hook knife	At the point of calf stiffness and irritation, pressure was applied and slid in the direction of the vertical muscle fibers	Low-Medium	1	Deep and precise relief of pain points in the lower leg

### Control group

In this study, the control group adopted the conventional training method, that is, ankle joint stability exercise and ankle muscle group strength training without equipment support. The training content of the control group was consistent with that of the experimental group, and the specific program is detailed in Table 1.

### Study outcomes

#### Cumberland ankle instability questionnaire

The Cumberland Ankle Instability Tool (CAIT) is an assessment tool designed to quantify a patient’s subjective perception of ankle instability (Hiller et al., 2006), including the frequency, intensity, and impact of symptoms on the patient’s life. The questionnaire was designed with a series of targeted questions, and each question was assigned a corresponding score according to the different answers. These scores are summed to form a total score that typically ranges from 0 to 30.

#### FAAM ankle function assessment questionnaire

The Foot and Ankle Ability Measure (FAAM) is a scale specifically used to evaluate the functional status of the ankle joint (Martin et al., 2005), which contains key questions for pain, function and quality of life, aiming to comprehensively evaluate the overall condition of patients with ankle instability. In terms of functional scores, FAAM includes two aspects: FAAM-ADL and FAAM-Sport. The scale is typically scored on a scale of 0–100, with a score of 100 indicating completely normal ankle function and a score of 0 indicating extremely limited ankle function or no use at all.

#### Ankle range of motion

In this study, a high-precision joint motion Angle measurement tool was used to strictly measure a variety of functional motion ranges of the ankle joint in sitting and supine positions. It specifically covers the range of dorsiflexion and plantarflexion of the ankle joint, and the range of varus and valgus of the foot (Winkelmann et al., 2016; Tian and Xu, 2005; Yang et al., 2019).

**FIGURE 3:**
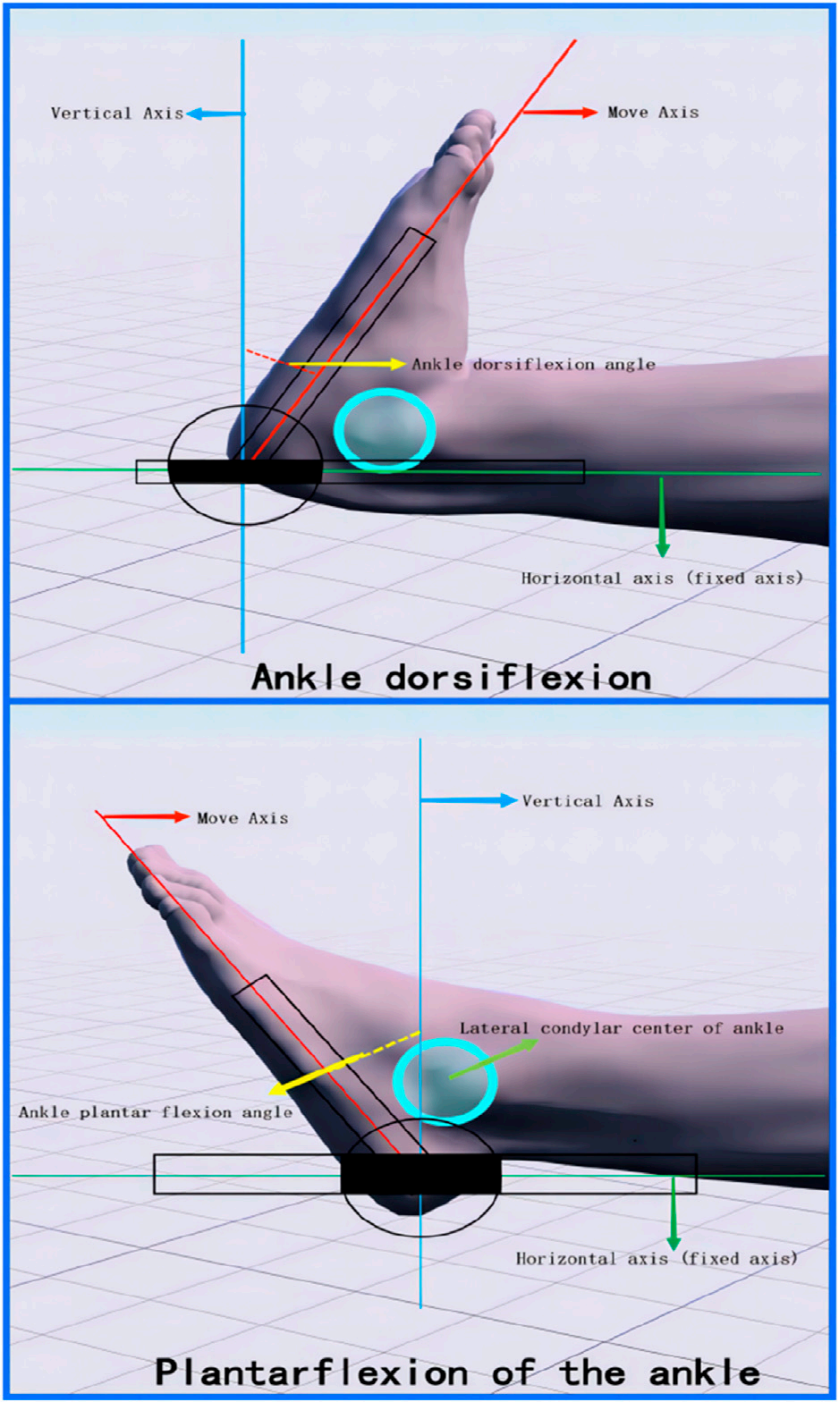
Range of motion measurements for ankle dorsiflexion *versus* plantarflexion.

Measurement of ankle dorsiflexion and plantar flexion (Figure 3). First, the subject was asked to lie flat on the edge of the treatment bed, ensuring that the knee joint was fully extended and the ankle joint was maintained in a natural neutral position. Subsequently, the center of the Angle measuring ruler was aligned with the side of the heel of the subject, and the fifth metatarsal bone was used as the longitudinal axis of movement. During the measurement, subjects were asked to start from a neutral position of the foot, lift the foot up to complete the flexion movement of the ankle joint, or step down to complete the extension movement of the ankle joint. The subjects should try their best to limit the amplitude of each exercise until they feel uncomfortable or reach the maximum comfort range. At this time, the Angle between the longitudinal axis of the ruler and the horizontal axis of the neutral position was measured, which was the range of motion of the ankle dorsiflexion or plantar flexion of the subject.

**FIGURE 4:**
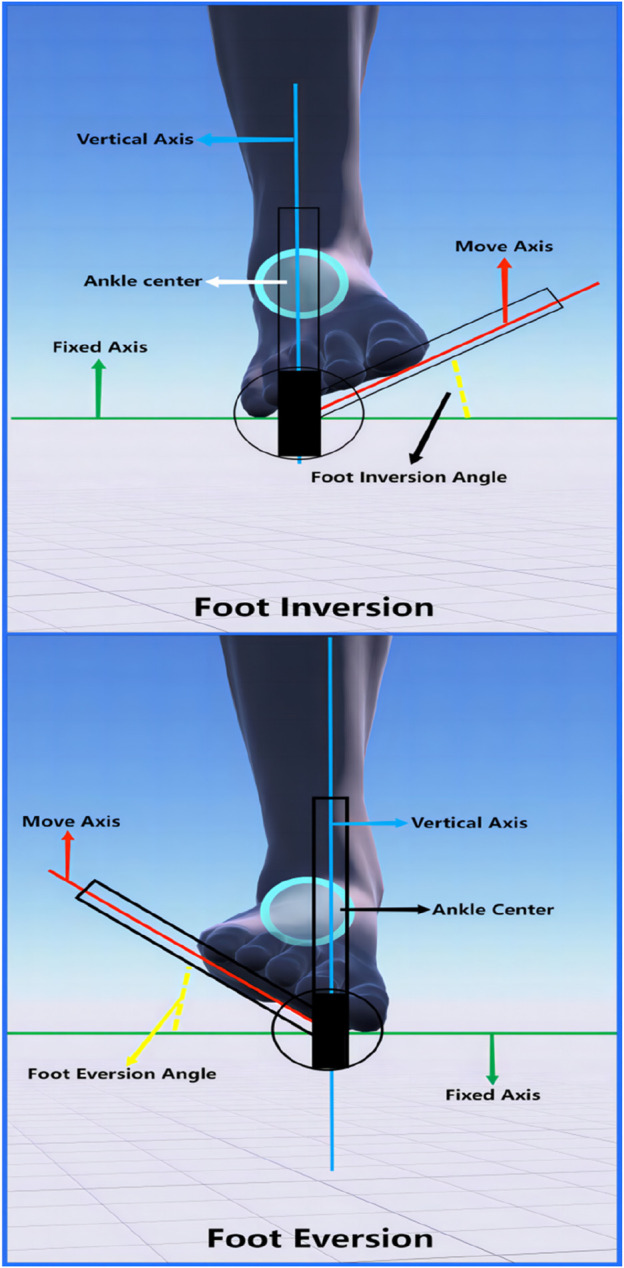
Range of motion measurements of the foot valgus versus varus.

Measurement of foot valgus and varus (Figure 4): Subjects should take a sitting position to ensure that the knee joint is naturally flexed and the ankle joint is kept in a neutral position. The fixed axis was defined as the longitudinal plantar axis perpendicular to the longitudinal axis of the calf, while the moving axis represented the moving plantar surface. The intersection of the two axes was defined as the axis. During the measurement, the movement of the outer edge of the foot of the subject up (foot valgus) or down (foot varus) was observed.

**FIGURE 5:**
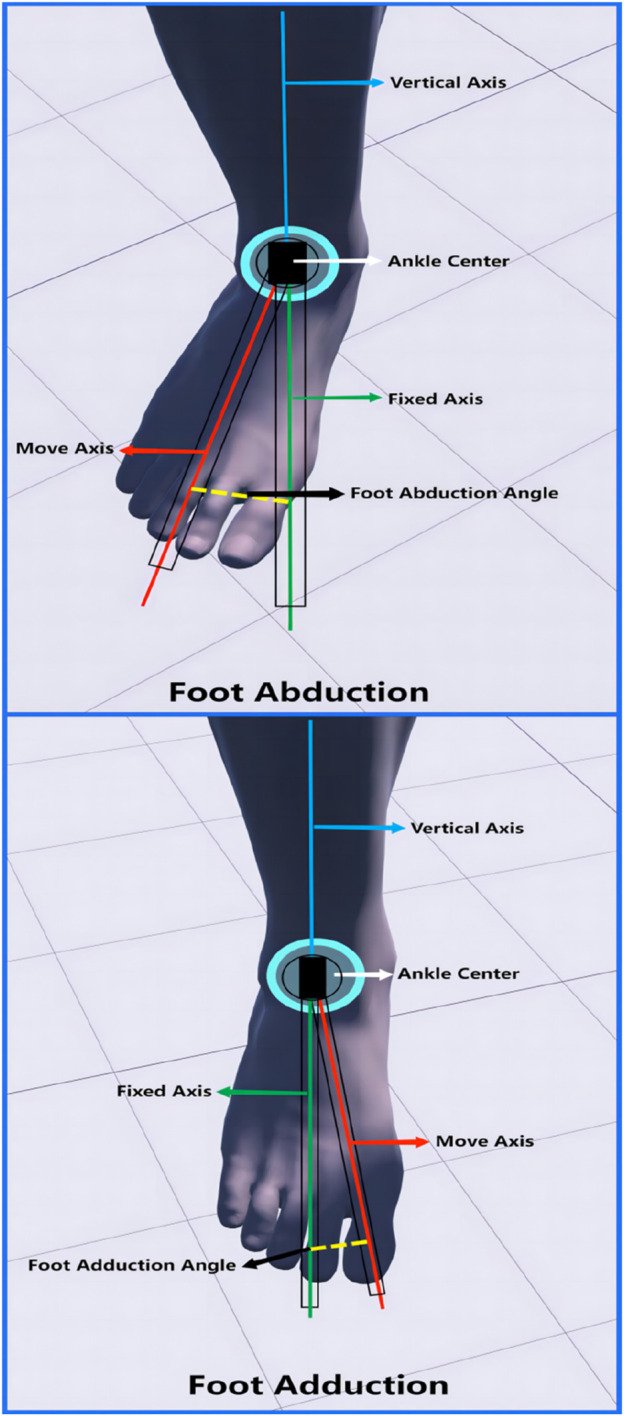
Measurement of range of motion of foot abduction and entrapment.

Standard procedure for foot abduction and adduction measurement (Figure 5): The subject should maintain a standing posture, ensuring that the knee joint is extended, the ankle joint is in a neutral position, and the lower leg is stable and still. The axis of measurement was set as the midpoint of the line connecting the anterior and medial condyle of the ankle and the lateral malleolus of the ankle. The fixation axis was the long axis of the foot between the first and second bones perpendicular to the axis. The moving axis refers to the changing foot length axis. When measurements were made, subjects were observed for movement of the lateral margin of the foot to the lateral side (foot abduction) or the medial margin of the foot to the medial side (foot adduction).

#### Ankle joint strength test

In this experiment, a handheld digital muscle strength tester (model FM-204 M series muscle strength tester) was used to measure strength data. The muscle strength tester measures in Newtons (N) and covers a measuring range of ±50 kg force (kgf), showing a measurement accuracy of ±0.5%FS (range) ±1 digital peak. Its measurable data include peak force and instantaneous force value. Therefore, the hand-held digital muscle strength tester was used in this study. The subjects were asked to sit in an appropriate position, ensure that the bottom of the instrument was stable on the ground, and then apply forces in different directions through the ankle. In the experiment, we focused on measuring the maximum strength in the dorsiflexion and plantar flexion of the ankle, as well as in the abduction and adduction directions of the foot. In order to ensure the accuracy and reliability of the data, the strength test of each different exercise session was repeated three times, and the average value of the three maximum strength values was finally taken as the final data.

#### Statistical analysis

The data for this study were analyzed and processed using the SPSS 26.0 statistical software package. The basic characteristics of the subjects were assessed using the independent samples *t*-test. For quantitative data that met the criteria for normal distribution, repeated measures analysis of variance (ANOVA) was employed for measurements taken at three time points. Paired t-tests were utilized for pre- and post-treatment measurements taken at two time points. A p-value of less than 0.05 was considered to indicate a statistically significant difference.

G.power 3.1.9.7 software was used to calculate the sample size. Due to the presence of repeated measures and interaction effects within the experimental groups in the analysis of variance, the software computation opts for F tests, specifically ANOVA: Repeated measures, between factors. For the type of power analysis, we have selected *A priori*: Compute required sample size, given the parameters of alpha, power, and effect size. The specific parameters include: Effect size f = 0.25, alpha error probability α = 0.05, Power (1-Beta error probability) = 0.8, Number of groups = 3, Number of measurements = 3, and Correlation among repeated measures = 0. The resultant sample size calculation is 54 divided by 3, which equals 18. However, the actual sample size for this experiment is 42 cases, which aligns with the results calculated by G. Power.

## Results

### Baseline data and recruitment results of subjects

**FIGURE 6:**
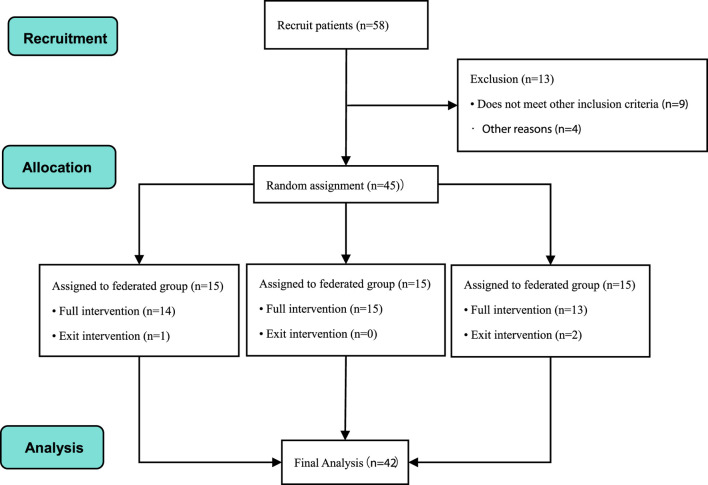
Flow chart of subject recruitment.

This experiment started the subject recruitment in Wuhan Institute of Physical Education. After strict inclusion criteria screening, 45 subjects who met the experimental requirements of sports dance were finally determined. However, by the fifth week of the experiment, three subjects chose to withdraw for personal reasons (1 in the combination group and 2 in the conventional group); therefore, the final data analysis was based on the results of only 43 subjects (see Figure 6 for details).

Participants were randomized into three groups: the combination group (BFRT combined with IASTM, n = 14), the BFRT alone group (n = 15), and the conventional ankle strength stabilization training group (n = 13). Detailed comparison of subjects’ information is shown in Table 3.

**TABLE 3:** Comparison of general data among the three groups.

Variable	Association group (n = 14)	BFRT (n = 15)	General group (n = 13)	F	P
Age (n)	20.21 ± 1.84	20.47 ± 1.64	19.38 ± 1.71	1.45	0.25
Gender (Male/Female)	8/6	4/11	7/6		
Height (Cm)	173.25 ± 9.15	168.93 ± 7.11	175.00 ± 7.62	1.07	0.35
Weight (Kg)	65.99 ± 24.20	61.53 ± 19.51	60.15 ± 9.76	0.35	0.71
Number Of Ankle Sprains In The Past Year (n)	1.43 ± 0.65	1.73 ± 0.59	1.62 ± 0.65	0.86	0.43
Affected Malleolar (n)					
Left	10	12	9		
Right	4	3	4		
Number Of Sprained Feet In Training (n)	2.50 ± 1.10	2.20 ± 1.01	2.23 ± 1.10	0.34	0.71
Ankle Sprain In The Past 5 Years (n)	3.00 ± 1.18	2.20 ± 1.01	2.23 ± 1.09	2.41	0.10
Yes	15	15			
No	0	0			
Specific Training Duration (Years)	6.71 ± 3.81	8.10 ± 4.56	6.23 ± 3.61	0.82	0.45

In this experiment, subjects recruitment and grouping, intervention implementation, data recording and statistical analysis were all completed independently by the first author. The basic information of the subjects included age, gender, height, weight, number of sprained feet, time engaged in special activities, etc. Through one-way ANOVA, the results showed that there was no significant difference in information among the subjects in each group (p > 0.05). For specific data, see Table 3.

### CAIT score results

ANOVA was conducted on the repeated measures data of the CAIT score at different time points, yielding the following conclusions: The effect of group changes on the CAIT score was not significant (F = 2.13, P = 0.13). However, the measurements at different time points significantly affected the CAIT score (F = 173.49, P < 0.05). The interaction between time points and groups also significantly influenced the CAIT score (F = 2.98, P = 0.02). Both the different time points and the interaction between groups and measurement time points significantly affected the changes in the subjects’ CAIT scores (Table 4).

**TABLE 4:** CAIT score table Multiple factor repeated measures ANOVA results.

CAIT repeated evaluation of the F-test			
	F	P	Bias η2
Group main effect	2.13	0.13	0.10
Time point main effect	173.49	0.00	0.82
Time point × Group	2.98	0.02	0.13

Within-group comparisons revealed that the CAIT scores for all three intervention groups significantly improved after the first intervention and at 6 weeks compared to pre-intervention (P < 0.05). The CAIT scores at 6 weeks post-intervention also showed a significant increase compared to those after the first intervention (P < 0.05) (Table 5).

**TABLE 5:** Results of comparison of changes in CAIT score table values with the mean values.

Grouping	Before	First	6-week	Multiple comparisons were made
	M±SD	M±SD	M±SD	
Combination	10.00 ± 1.75	16.64 ± 2.41*and	26.14 ± 3.21*and**#**	Before < First < 6-Week
BFRT	11.81 ± 2.80	19.73 ± 5.37*and	24.93 ± 3.31*and	Before < First < 6-Week
Conventional	11.38 ± 2.59	18.08 ± 4.89*and	22.54 ± 2.33*and**#**	Before < First < 6-Week

Note; * There was a significant difference in CAIT, score between before and after intervention (p < 0.05). and represents a significant change after the first intervention and 6 weeks after the intervention (p < 0.05). **#** represents the significant change between different intervention groups (p < 0.05). M±SD, represents the mean ± standard deviation.

When comparing between groups, only the combined group and the conventional group exhibited significant differences after 6 weeks of intervention (P < 0.05). For other groups and time points, the changes among the three intervention groups were not significant (P > 0.05). Observing the mean CAIT scores after 6 weeks of intervention, the combined group scored higher than the other two groups. In summary, all three intervention groups effectively improved the instability of the patients’ ankle joints, with the combined group showing a more pronounced effect.

### FAAM ankle function evaluation results

The FAAM scale focuses on collecting data closely related to ankle function, which mainly covers two aspects. The first is FAAM-ADL score, which focuses on assessing the ankle function of patients in daily life. The second is the FAAM-SPORT scale, which focuses on measuring the ankle function of patients when participating in sports activities.

### FAAM-ADL score

The variation in groups had no significant effect on the FAAM-ADL scores (F = 1.96, P = 0.16), while the measurements at different time points significantly influenced the FAAM-ADL scores (F = 272.26, P < 0.05). The interaction between time points and groups did not significantly affect the FAAM-ADL scores (F = 0.18, P = 0.93). In summary, only the factor of measurements at different time points had a significant impact on the FAAM-ADL scores (Table 6).

**TABLE 6:** FAAM-ADL score table Multiple factor repeated measures analysis of variance results.

FAAM-ADL repeated evaluation F test			
	F	P	Bias η2
Group main effect	1.96	0.16	0.09
Time point main effect	272.26	0.00	0.88
Time point × Group	0.18	0.93	0.01

Within-group comparisons showed that the FAAM-ADL scores for all three intervention groups were higher after the initial intervention and at the 6-week intervention compared to pre-intervention (P < 0.05), and the scores after 6 weeks of intervention were also higher than those after the initial intervention (P < 0.05). When comparing between different groups, only the BFRT group and the control group exhibited significant differences after 6 weeks of intervention (P < 0.05), with the BFRT group having a notably higher mean FAAM-ADL score than the control group.

The results indicate that all three intervention groups facilitated an improvement in ankle joint function in daily activities for the patients, with the BFRT group showing a more pronounced therapeutic effect (Table 7).

**TABLE 7:** Comparison results of FAAM-ADL score table value changes and mean values.

Grouping	Before	First	6-week	Multiple comparisons were made
	M±SD	M±SD	M±SD	
Combination	41.79 ± 16.13	82.86 ± 11.39*&	93.57 ± 6.02*&	Before < First < 6-Week
BFRT	40.67 ± 12.80	83.67 ± 11.26*&	96.00 ± 4.31*&	Before < First < 6-Week
Conventional	38.46 ± 17.25	77.69 ± 6.33*&	90.00 ± 7.07*&	Before < First < 6-Week

Note; * There was a significant difference in CAIT, score between before and after intervention (p < 0.05). & represents a significant change after the first intervention and 6 weeks after the intervention (p < 0.05). # represents the significant change between different intervention groups (p < 0.05). M±SD, represents the mean ± standard deviation.

### FAAM-SPORT score

The effect of group changes on FAAM-SPORT scores was not significant (F = 3.23, P = 0.05). However, the impact of measurements at different time points on FAAM-SPORT scores was highly significant (F = 390.46, P < 0.05). The interaction effect between time points and groups on FAAM-SPORT scores was not significant (F = 4.93, P = 0.39). Consequently, different group assignments did not significantly affect the participants’ FAAM-SPORT scores, while measurements at different time points and the interaction between group assignments and measurement time points had a significant impact on FAAM-SPORT scores (Table 8).

**TABLE 8:** FAAM-SPORTL score table Multiple factor repeated measures ANOVA results.

FAAM-SPORT repeated evaluation F test			
	F	P	Bias η2
Group main effect	3.23	0.05	0.14
Time point main effect	390.46	0.00	0.91
Time point × Group	1.01	0.39	0.04

Within-group comparative analysis revealed that after the initial and 6-week interventions, the FAAM-SPORT scores for all three intervention groups were significantly higher than pre-intervention levels (P < 0.05). The combined group and BFRT group also showed a significant improvement in FAAM-SPORT scores after the 6-week intervention compared to the initial intervention (P < 0.05). The results indicate that the 6-week intervention further improved the patients’ ankle joint performance in sports activities. In contrast, the control group did not exhibit significant changes in FAAM-SPORT scores after the initial and 6-week interventions (P > 0.05).

When comparing between different groups, it was noted that the FAAM-SPORT scores for the combined group and BFRT group after the initial intervention were significantly higher than those of the control group (Table 9). The interventions of the combined group and BFRT group were effective in enhancing the patients’ performance in sports activities immediately after the first implementation.

**TABLE 9:** Results of comparison between FAAM-SPORT score table value changes and mean values.

Grouping	Before	First	6-week	Multiple comparisons were made
	M±SD	M±SD	M±SD	
Combination	32.50 ± 11.22	80.71 ± 10.54*&	95.71 ± 4.32*&	Before < First < 6-Week
BFRT	28.67 ± 15.98	78.67 ± 9.16*&	92.00 ± 6.76*&	Before < First < 6-Week
Conventional	34.62 ± 15.06	87.69 ± 5.63*	91.92 ± 5.60*	Before < First < 6-Week

Note; * There was a significant difference in CAIT, score between before and after intervention (p < 0.05). & represents a significant change after the first intervention and 6 weeks after the intervention (p < 0.05). # represents a significant change compared with the conventional group (p < 0.05). M±SD, represents the mean ± standard deviation.

### Measurement results of range of motion of the lower ankle

In this study, we systematically evaluated the ankle range of motion (ROM) of CAI patients in the sitting and supine positions using high-precision measuring devices. The assessment included ankle dorsiflexion and plantarflexion angles, foot varus and valgus angles, and foot abduction and adduction angles. The purpose of this study is to accurately understand the specific condition of ankle dysfunction in CAI patients, so as to provide scientific and reliable basis for subsequent rehabilitation treatment.

### Dorsiflexion and plantar flexion

In a supine position, measurements of dorsal flexion and plantar flexion angles of the foot were taken and compared using repeated measures analysis of variance (ANOVA) for data collected pre-intervention, post-initial intervention, and at 6 weeks post-intervention. The results indicated that group changes had a significant effect on dorsal flexion angles (F = 13.30, P < 0.05), while no significant effect was observed for plantar flexion angles (F = 1.67, P = 0.20). Measurements at different time points significantly affected both dorsal and plantar flexion angles (F = 677.64, P < 0.05; F = 123.34, P < 0.05). The interaction between time points and groups also significantly influenced the measurements of dorsal and plantar flexion angles (F = 113.42, P < 0.05; F = 59.05, P < 0.05). In summary, both different time points and the interaction between the two factors significantly affected the results of the dorsal and plantar flexion angle measurements (Table 10).

**TABLE 10:** Results of multiple factor repeated measures ANOVA for dorsiflexion and plantarflexion angles.

	Dorsiflexion			Plantar flexion		
	F	P	Bias η2	F	P	Bias η2
Group main effect	13.30	0.00	0.41	1.67	0.20	0.08
Time point main effect	677.64	0.00	0.95	123.34	0.00	0.76
Time point × Group	113.42	0.00	0.85	59.05	0.00	0.75

In within-group comparisons, all three intervention groups showed a significant increase in dorsal flexion angle measurements after the initial and 6-week interventions (P < 0.05). The dorsal flexion angle measurements at 6 weeks post-intervention were significantly higher than those post-initial intervention (P < 0.05). For plantar flexion angle measurements, only the combined group showed significant effects at the three different time points. The BFRT group had higher plantar flexion angle measurements after the initial and 6-week interventions compared to pre-intervention (P < 0.05), while the control group only showed a significant increase in plantar flexion angle at 6 weeks post-intervention compared to pre-intervention (P < 0.05). This suggests that the combined group effectively enhanced the range of motion for dorsal and plantar flexion of the foot both immediately after treatment and at 6 weeks post-treatment; whereas the BFRT group and the control group mainly improved the ankle dorsal flexion and plantar flexion range of motion significantly after the 6-week short-term treatment. Immediate treatment did not significantly enhance the range of motion of the ankle joint, especially as the control group did not show a significant immediate effect on improving the plantar flexion angle of the ankle joint.

In between-group comparisons, the combined group had a significant advantage in dorsal flexion angle after both the initial and 6-week interventions compared to the BFRT group and the control group. Additionally, the combined group also showed a clear advantage in plantar flexion angle at 6 weeks post-intervention compared to the BFRT group and the control group. The results suggest that the BFRT combined with IASTM can significantly improve the range of motion for dorsal and plantar flexion of the foot in patients with chronic ankle instability in the short term, with better outcomes than simple BFRT and conventional ankle joint strength stabilization training (Table 11).

**TABLE 11:** Comparison results of the changes in the Angle values of the range of motion between dorsiflexion and plantarflexion and the mean values.

Grouping	Project/Angle	Before	First	6-week	Multiple comparisons were made
		M±SD	M±SD	M±SD	
Combination (n = 14)	Dorsiflexion	15.59 ± 2.70	23.90 ± 4.31*&	37.51 ± 2.98*&	Before < First < 6-Week
	Plantar flexion	38.17 ± 7.08	46.19 ± 5.13*&	62.07 ± 7.08*&	Before < First < 6-Week
BFRT (n = 15)	Dorsiflexion	16.03 ± 3.02	19.93 ± 3.02*#&	25.36 ± 4.08*#&	Before < First < 6-Week
	Plantar flexion	40.92 ± 6.71	47.48 ± 9.22*	45.87 ± 7.44*#	Before < First < 6-Week
Conventional (n = 13)	Dorsiflexion	17.38 ± 3.30	19.98 ± 3.30*#&	22.77 ± 3.92*#&	Before < First < 6-Week
	Plantar flexion	47.15 ± 5.94**#a**	49.53 ± 8.22	49.76 ± 8.24*#	Before < First < 6-Week

Note: * represents the significant difference in dorsiflexion Angle between before and after intervention (p < 0.05); & represents a significant change after the first intervention and 6 weeks after the intervention (p < 0.05). Compared with the combined group, # represented a significant change (p < 0.05). A represents a significant change when compared with BFRT, group (p < 0.05); M±SD, represents the mean ± standard deviation.

### Varus and valgus

Measurements of inversion and eversion angles in a seated position revealed that changes in group assignment had a significant effect on the inversion angle (F = 11.41, P < 0.05), but not on the eversion angle (F = 0.52, P = 0.59). Measurements at different time points significantly influenced both inversion and eversion angles (F = 96.38, P < 0.05; F = 31.46, P < 0.05). The interaction between time points and group assignment also significantly affected the measurements of inversion and eversion angles (F = 19.13, P < 0.05; F = 6.52, P < 0.05). The results indicate that different group assignments, different time points, and the interaction between these two factors (group assignment and measurement time points) significantly influenced the measurement of the inversion angle (Table 12).

**TABLE 12:** Results of multiple factor repeated measures ANOVA for varus and valgus angles.

	Varus			Valgus		
	F	P	Bias η2	F	P	Bias η2
Group main effect	11.41	0.00	0.37	0.52	0.59	0.02
Time point main effect	96.38	0.00	0.71	31.46	0.00	0.45
Time point × Group	19.13	0.00	0.50	6.52	0.00	0.25

Within-group comparative analysis showed that after the initial and 6-week interventions, there was a significant increase in the inversion angle for all three intervention groups (P < 0.05). Only the combined group exhibited a significant improvement in the inversion angle after 6 weeks compared to the initial intervention (P < 0.05). In the assessment of the eversion angle, the combined group showed a significant effect at all three time points. Additionally, the BFRT group showed an increase in the plantar flexion angle after 6 weeks of intervention compared to pre-intervention (P < 0.05), while the control group did not show significant changes in all three measurements (P > 0.05). In summary, the combined group effectively expanded the range of motion for foot inversion and eversion both immediately and after short-term treatment; the BFRT group only demonstrated a significant enhancement in the range of motion for foot inversion and eversion after short-term treatment; and at the immediate treatment stage, the improvement in the range of motion of the ankle joint was not significant in all groups, especially the control group, which did not show a clear effect in promoting the range of motion for ankle inversion and eversion.

Between-group comparisons revealed that the combined group had significantly higher inversion angles after the initial intervention and after 6 weeks of intervention compared to the BFRT group and the control group. Therefore, the combination of BFRT and IASTM can effectively enhance the range of motion for foot inversion and eversion in patients with CAI in the short term, with more pronounced therapeutic effects than simple BFRT and conventional ankle joint strength stabilization training (Table 13).

**TABLE 13:** Results of comparison of varus and valgus Angle numerical changes and mean values.

Grouping	Project/Angle	Before	First	6-week	Multiple comparisons were made
		M±SD	M±SD	M±SD	
Combination (n = 14)	Varus	31.77 ± 6.43	42.55 ± 7.08*&	54.72 ± 6.41*&	Before < First < 6-Week
	Valgus	16.18 ± 3.33	17.62 ± 3.38*&	20.80 ± 5.29*&	Before < First < 6-Week
BFRT (n = 15)	Varus	31.61 ± 5.81	37.51 ± 6.20*#	39.32 ± 6.95*#	Before < First < 6-Week
	Valgus	15.84 ± 3.21	16.29 ± 3.08&	18.85 ± 3.45*&	Before < First < 6-Week
Conventional (n = 13)	Varus	30.80 ± 4.35	34.90 ± 4.31*#	36.79 ± 6.14*#	Before < First < 6-Week
	Valgus	17.23 ± 3.45	16.67 ± 3.38	17.43 ± 4.34	Before > First < 6-Week

Note: * represents the significant difference in dorsiflexion Angle between before and after intervention (p < 0.05); & represents a significant change after the first intervention and 6 weeks after the intervention (p < 0.05). Compared with the combined group, # represented a significant change (p < 0.05). M±SD, represents the mean ± standard deviation.

### Adduction and abduction

The effect of group changes on the adduction angle was not significant (F = 0.06, P = 0.80), while the effect on the abduction angle was significant (F = 9.80, P < 0.05). Measurements at different time points significantly influenced both the adduction and abduction angles (F = 96.33, P < 0.05; F = 939.10, P < 0.05). The interaction between time points and groups did not significantly affect the measurement of the adduction angle (F = 14.17, P < 0.05), but it did significantly influence the abduction angle (F = 22.19, P < 0.05). It can be concluded that different time points and the interaction between the two factors (group assignment and measurement time points) significantly affected the measurements of both adduction and abduction angles (Table 14).

**TABLE 14:** Adduction and abduction multiple factor repeated measures ANOVA results.

	Adduction			Abduction		
	F	P	Bias η2	F	P	Bias η2
Group main effect	0.06	0.80	0.00	9.80	0.00	0.33
Time point main effect	96.33	0.00	0.71	939.10	0.00	0.96
Time point × Group	14.17	0.00	0.42	22.19	0.00	0.53

Within-group comparisons revealed that all three intervention groups showed a significant increase in both adduction and abduction angles after the initial intervention and at 6 weeks (P < 0.05). Only the combined group and the BFRT group exhibited a more pronounced improvement in adduction and abduction angles after 6 weeks compared to the initial intervention (P < 0.05). Therefore, both the combined group and the BFRT group effectively enhanced the range of motion for ankle joint adduction and abduction, whether immediately after treatment or after short-term treatment. In contrast, the control group only significantly increased the ankle joint adduction and abduction angles after 6 weeks of short-term treatment.

Between-group comparisons indicated that the combined group had significantly higher ankle joint adduction and abduction angles after the initial intervention and at 6 weeks compared to the BFRT group and the control group. It can be concluded that the combination of BFRT and IASTM significantly improved the range of motion for ankle joint adduction and abduction in patients with CAI in the short term, with more pronounced effects than simple BFRT and conventional ankle joint strength stabilization training (Table 15).

**TABLE 15:** Adduction and abduction angle numerical changes and mean comparison results.

Grouping	Project/Angle	Before	First	6-week	Multiple comparisons were made
		M±SD	M±SD	M±SD	
Combination (n = 14)	Adduction	28.25 ± 3.84	33.55 ± 4.18*&	37.29 ± 4.91*&	Before < First < 6-Week
	Abduction	23.40 ± 4.01	49.58 ± 5.93*&	51.62 ± 3.17*&	Before < First < 6-Week
BFRT (n = 15)	Adduction	27.11 ± 3.75	28.17 ± 2.80*&**#**	31.64 ± 3.82*&**#**	Before < First < 6-Week
	Abduction	23.46 ± 4.16	40.92 ± 3.47*&**#**	42.78 ± 3.34*&**#**	Before < First < 6-Week
Conventional (n = 13)	Adduction	26.38 ± 3.45	28.69 ± 3.34*#	28.96 ± 3.69*#	Before < First < 6-Week
	Abduction	25.50 ± 3.89	41.74 ± 5.78*#	43.04 ± 3.96*#	Before < First < 6-Week

Note: * represents the significant difference in dorsiflexion Angle between before and after intervention (p < 0.05); & represents a significant change after the first intervention and 6 weeks after the intervention (p < 0.05). Compared with the combined group, # represented a significant change (p < 0.05). M±SD, represents the mean ± standard deviation.

### Ankle joint strength test

This experiment utilized a hand-held dynamometer to precisely measure the maximum strength of patients with CAI in various functional states of the ankle joint. The measurement process encompassed multiple dimensions of movement strength, including dorsal flexion, plantar flexion, adduction, and abduction of the ankle joint.

Within-group comparative analysis revealed that after intervention, all three intervention groups exhibited significant increases in the strength of ankle joint movements, including dorsal flexion, plantar flexion, adduction, and abduction (P < 0.05). The three distinct exercise intervention programs were all effective in enhancing the ankle joint strength levels of patients with CAI. When comparing data between different groups, it was found that the combined group showed significant improvements in dorsal flexion and adduction strength of the ankle joint compared to the control group after intervention (P < 0.05). In other aspects of ankle joint movement strength, there were no statistically significant differences between the groups (*p* > 0.05) (See Table 16).

**TABLE 16:** Comparison of mean values of ankle joint strength for different functions in standing posture.

Grouping	Project/Power	Before/N	6-week/N	Multiple comparisons were made
		M±SD	M±SD	
Combination (n = 14)	Dorsiflexion	43.92 ± 20.70*	160.82 ± 25.92*a	Before < 6-Week
	Plantar Flexion	94.06 ± 16.88*	149.12 ± 25.57*	Before < 6-Week
BFRT (n = 15)	Adduction	31.85 ± 10.47*	68.98 ± 13.96*a	Before < 6-Week
	Abduction	51.49 ± 12.79*	86.80 ± 19.55*	Before < 6-Week
	Dorsiflexion	42.73 ± 14.35*	145.82 ± 30.40*	Before < 6-Week
	Plantar Flexion	96.05 ± 18.96*	151.56 ± 25.84*	Before < 6-Week
	Adduction	30.79 ± 12.45*	60.49 ± 16.44*	Before < 6-Week
	Abduction	49.68 ± 12.57*	78.50 ± 19.18*	Before < 6-Week
Conventional (n = 13)	Dorsiflexion	52.26 ± 14.60*	131.42 ± 35.97*a	Before < 6-Week
	Plantar Flexion	87.67 ± 17.07*	136.76 ± 26.64*	Before < 6-Week
	Adduction	32.92 ± 11.40*	55.70 ± 14.93*a	Before < 6-Week
	Abduction	52.84 ± 13.53*	78.09 ± 19.94*	Before < 6-Week

Note: * represents the significant difference in the change of the mean strength before and after the intervention in the intra-group comparison (p < 0.05); a represents the significant difference in the mean change of strength between groups (p < 0.05).

## Discussion

This study explored the application of ankle blood flow restriction training combined with IASTM in patients with dancesit-related CAI. The results showed that after 6 weeks of intervention, three intervention methods could effectively improve the ankle stability of CAI patients. The effect of the combined group in improving the stability of the ankle joint is significantly better than that of the BFRT group and the conventional group, showing its unique advantages and application value. According to the results of FAAM functional score, all three intervention groups had a significant promotion effect on the ankle joint function of CAI patients. In terms of FAAM-ADL score, which assesses ankle movement ability in daily life, all three intervention groups showed positive effects, and the BFRT group had a particularly significant effect. In the FAAM-SPORT score for motor function, the combined group and the BFRT group performed better than the conventional group after the first intervention, which fully proved that these two groups of interventions could have a significant positive effect on the motor function of CAI patients after the first treatment. After comprehensive evaluation of the recovery of the range of motion of the ankle joint in the three groups of CAI patients, it was found that there was a certain degree of improvement. The ankle dorsiflexion, abduction, varus, adduction and abduction angles of the combined group were significantly improved after the first intervention and 6 weeks after the intervention, especially in the improvement of foot dorsiflexion ability. In contrast, the BFRT group and the conventional group showed significant effects only after 6 weeks of short-term treatment, and there was no significant improvement in the adduction-valgus ability of the ankle joint. In terms of ankle strength improvement, it was found that all three intervention groups showed positive effects in improving ankle strength, which was manifested in the improvement of dorsiflexion, plantarflexion, adduction and abduction. In particular, the improvement of ankle dorsiflexion and adduction strength in the combined group was significantly greater than that in the conventional group.

It can be concluded that all three intervention groups can improve the symptoms of CAI athlete patients to some extent. However, compared with the other control groups, the combination group was more effective in expanding the range of motion and enhancing the strength of the ankle, especially when compared with the conventional ankle joint strength and stability training.

CAI is associated with collateral ligament injuries, especially the tibial collateral ligament (Bonnel et al., 2010). Repeated sprains or improper rehabilitation may lead to ligament relaxation and loss of support for the ankle joint, increasing the risk of excessive displacement and thus aggravating joint instability (Bonnel et al., 2010). This is one of the important causes of ankle injuries in sports dancers. In addition, habitual sprains may result in sensory nerve abnormalities that attenuate the ability of athletes to perceive joint position. This further leads to the inability to accurately perceive joint position during movement, which significantly increases the risk of re-injury (Hansen et al., 2022). At the same time, long-term ligament injury may also trigger motor control disorders, including muscle coordination and balance problems. These dysfunctions make it difficult to precisely control the ankle during daily activities and sports, further increasing the risk of injury (Miklovic et al., 2018). Habitual sprain may lead to sensory nerve abnormalities, weaken the athlete’s ability to perceive the joint position, and increase the risk of re-injury (Hansen et al., 2022). In addition, long-term ligament injury may also cause motor control disorders, including muscle coordination and balance problems, further increasing the risk of injury (Miklovic et al., 2018).

Chronic ligament injury and laxity affect ankle stability and lead to an increased risk of abnormal displacement. Damaged ligaments can cause adaptive changes in the surrounding soft tissues, such as muscle atrophy and morphological changes of tendons, further weakening structural support and aggravating functional damage. This study combined the special mechanism of BFR with the release treatment of IASTM, aiming to improve the symptoms of patients with CAI at multiple levels. This method is expected to provide a comprehensive treatment plan for CAI patients and promote the stability and functional recovery of the ankle joint.

BFRT promotes muscle growth mainly through metabolic and mechanical tension mechanisms. The use of pneumatic bands to limit blood return creates a hypoxic environment, increases lactic acid accumulation in muscle (Saatmann et al., 2021), triggers a metabolic response, releases growth hormone, and promotes muscle growth. At the same time, BFRT restricts blood flow, reduces muscle oxygen supply, causes glyoxylate accumulation, stimulates the neuromuscular system, enhances muscle mechanical tone, and achieves an increase in muscle strength and volume under low load (Caetano et al., 2021). Therefore, by limiting the blood flow of lower limb muscles and reducing the oxygen supply of muscles in sports dance athletes, BFRT stimulates the sensation of lower limb muscles in the neuromuscular system, enhances the muscle strength around the ankle joint, and achieves high-intensity training effect with low load. At the same time, it promotes the muscle groups that stabilize the ankle joint, improves neuromuscular control, and ultimately improves ankle stability.

IASTM is widely used in the field of physical therapy, and its combination with other physical therapy methods such as cold and hot compress and electrical stimulation can enhance the therapeutic effect (Lu et al., 2020; Mylonas et al., 2021). In rehabilitation medicine, IASTM is widely used to treat various types of sports injuries, including muscle strains and ligament injuries. In the field of sports medicine, IASTM technology can help athletes recover quickly and improve their sports performance (Liu and Wu, 2024). During the treatment, the therapist used the IASTM tool to scrub the patient’s soft tissue to release the adhesion tissue and promote blood circulation (Gunn, et al., 2019). At the same time, the therapist adjusts the pressure of the tool and the intensity of scraping according to the feedback of the patient to ensure the comfort and effectiveness of the treatment. IASTM technology can not only help athletes maintain a healthy sports state by preventing and treating sports injuries, but also show significant application potential in the fields of rehabilitation medicine, sports medicine, and plastic surgery. Stanek et al. (2018) studied that IASTM can significantly improve the flexion limitation of the ankle joint and the range of motion of the ankle joint. In addition, a large number of studies have shown that IASTM can increase the ability of joint range of motion and improve the pain of patients in a short period of time, which is a worthy physical therapy method in clinical treatment (Seffrin et al., 2019; França et al., 2023; Cheatham et al., 2016).

Overall, IASTM demonstrated significant applicability in the treatment of CAI symptoms. This method can effectively adjust the range of motion of the ankle joint, stimulate the surface of soft tissue through professional tools, induce local inflammatory response, and then promote blood circulation and nerve function regulation, significantly relieve pain, improve motor dysfunction, and help patients recover. This study showed that the combined application of BFRT and IASTM has a significant effect on patients with CAI in the field of sports dance.

According to the study by Deodato et al. (2022), an assessment method based on inertial sensors revealed that during single-leg standing, the “healthy” limb of patients with chronic ankle instability may not be entirely healthy. This finding indicates that even the uninjured limb may undergo a reorganization of the sensorimotor system due to injury on the contralateral side, thereby affecting postural control. Further research by Groters et al. (2013) has indicated that individuals with functional ankle instability also have issues with double-leg standing and dynamic balance. This suggests that an injury to a single ankle joint may have broad implications for the body’s overall balance and motor control. Based on these studies, the importance of considering both ankles in the assessment and treatment of chronic ankle instability is recognized. While the present study primarily focuses on the intervention effects on the injured ankle, the aforementioned research suggests that future studies should more comprehensively account for the functional status and interplay of both ankles.

However, the current study has certain limitations, including a small sample size of patients with CAI, a brief treatment period, and a focus primarily on athletic populations. These factors may introduce selection bias and limitations in the interpretation of results.

Future research should aim to enlarge the sample size, encompassing a more diverse population, and conduct long-term follow-ups post-treatment. It should also delve deeper into the physiological mechanisms underlying Chronic Ankle Instability (CAI). Advanced investigations could involve the use of electroencephalography (EEG) and electromyography (EMG) to monitor the neurological and muscular activities associated with motor control or postural control in CAI patients. Such assessments could yield a comprehensive understanding of the synergistic mechanisms of neuromuscular function in individuals with CAI. Evaluating the mechanisms of action and efficacy of various therapeutic exercises may facilitate the precision design of tailored exercise intervention programs, enhancing the personalization of treatment strategies for CAI patients.

## Data Availability

The original contributions presented in the study are included in the article/supplementary material, further inquiries can be directed to the corresponding author.
